# *N*′-(Phenyl­sulfon­yl)isonicotinohydrazide monohydrate

**DOI:** 10.1107/S1600536809016651

**Published:** 2009-05-14

**Authors:** Chun-Rong Li, Kai-Zhi Zhou, Yun-Qian Zhang, Sai-Feng Xue, Hang Cong

**Affiliations:** a Key Laboratory of Macrocyclic and Supramolecular Chemistry, of Guizhou Province, Guizhou University, Guiyang, 550025, People’s Republic of China.

## Abstract

In the title compound, C_12_H_11_N_3_O_3_S·H_2_O, the pyridine ring makes a dihedral angle of 24.78 (14)° with the phenyl ring. Intra­molecular N—H⋯O and inter­molecular O—H⋯O hydrogen bonds are observed and stabilize the packing in the crystal structure.

## Related literature

For general background to hydrazide derivatives, see: Lemin (1961 ▶); Shanbhag *et al.* (2008 ▶); Zhen & Li (2008 ▶).
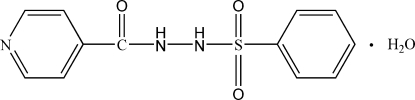


## Experimental

### 

#### Crystal data

C_12_H_11_N_3_O_3_S·H_2_O
*M* *_r_* = 295.32
Monoclinic, 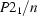

*a* = 7.3525 (5) Å
*b* = 20.9324 (15) Å
*c* = 9.2443 (6) Å
β = 107.565 (2)°
*V* = 1356.41 (16) Å^3^
*Z* = 4
Mo *K*α radiation
μ = 0.26 mm^−1^
*T* = 273 K
0.24 × 0.22 × 0.19 mm

#### Data collection

Bruker SMART APEX CCD area-detector diffractometer
Absorption correction: multi-scan (*SADABS*; Bruker, 2005 ▶) *T*_min_ = 0.833, *T*_max_ = 0.864 (expected range = 0.918–0.953)
10653 measured reflections
2343 independent reflections
1981 reflections with *I* > 2σ(*I*)
*R*_int_ = 0.033

#### Refinement

*R*[*F* ^2^ > 2σ(*F* ^2^)] = 0.037
*wR*(*F* ^2^) = 0.120
*S* = 1.12
2343 reflections
189 parameters
H atoms treated by a mixture of independent and constrained refinement
Δρ_max_ = 0.43 e Å^−3^
Δρ_min_ = −0.53 e Å^−3^

### 

Data collection: *SMART* (Bruker, 2002 ▶); cell refinement: *SAINT* (Bruker, 2002 ▶); data reduction: *SAINT*; program(s) used to solve structure: *SHELXS97* (Sheldrick, 2008 ▶); program(s) used to refine structure: *SHELXL97* (Sheldrick, 2008 ▶); molecular graphics: *ORTEP-3 for Windows* (Farrugia, 1997 ▶); software used to prepare material for publication: *WinGX* (Farrugia, 1999 ▶).

## Supplementary Material

Crystal structure: contains datablocks global, I. DOI: 10.1107/S1600536809016651/at2777sup1.cif

Structure factors: contains datablocks I. DOI: 10.1107/S1600536809016651/at2777Isup2.hkl

Additional supplementary materials:  crystallographic information; 3D view; checkCIF report

## Figures and Tables

**Table 1:** Hydrogen-bond geometry (Å, °)

*D*—H⋯*A*	*D*—H	H⋯*A*	*D*⋯*A*	*D*—H⋯*A*
N3—H3*N*⋯O1*W*	0.86	2.04	2.779 (3)	144
O1*W*—H1*E*⋯O2^i^	0.78 (4)	2.07 (4)	2.857 (3)	175 (4)

Symmetry code: (i) 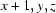.

**Fig. 1.**
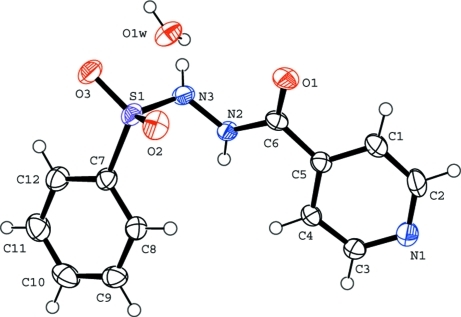
The molecular structure of (I) showing the atom-labelling scheme. Displacement ellipsoids are drawn at the 50% probability level.

## supplementary crystallographic information

### Comment

Hydrazide derivatives investigated in the present work are non-toxic in nature, which play an important role in latex, plastic industry (Lemin *et al.*, 1961; Zhen *et al.*, 2008) and corrosion inhibition of mild steel in acidic medium (Shanbhag *et al.*, 2008). In this paper, a substituted hydrazide, benzenesulfisoniazide, was synthesized in the solution of ethanol with benzenesulfonyl chloride and isoniazide in the ice-bath is reported.

The crystal of the title compound, (I), consists of *N*'-(phenylsulfinyl)isonicotinohydrazide and one water molecule, (Fig. 1). Pyridine ring makes a dihedral angle of 24.78 (14)° with the phenyl ring. The N—H···O hydrogen bonds are observed between N3 and the water molecule O1W, which the distance of the N3(H3N)···O1W hydrogen bonds is 2.779 (3) Å. In addition, there are O—H···O hydrogen bonds between O1W and O2 with distance of 2.857 (3) Å (Table 1). These hydrogen bonding interactions may help to establish the packing in the crystal structure.

### Experimental

Solution of benzenesulfonyl chloride (0.04 mol) in ethanol was added to a stirred ethanol solution of isoniazid (0.02 mol) in the ice-bath, then the reaction was kept on for 2 h at room temperature. The solvent was removed by reduced pressure filter, the solid product was dissolved in 50 ml ethanol,. and then set aside for five days to obtain colourless crystals.

### Refinement

Water H atoms were located in a difference Fourier map and refined as riding in their as-found positions relative to O atoms with *U*_iso_(H) = 1.5*U*_eq_(O). All other H atoms were placed in calculated positions and refined as riding, with C—H = 0.93–0.97 Å, N—H = 0.86 Å, and *U*_iso_(H) = 1.2–1.5 *U*_eq_(C,*N*).

### Crystal data

**Table:**

C_12_H_11_N_3_O_3_S·H_2_O	*F*(000) = 616
*M**_r_* = 295.32	*D*_x_ = 1.446 Mg m^−^^3^
Monoclinic, *P*2_1_/*n*	Mo *K*α radiation, λ = 0.71073 Å
Hall symbol: -P 2yn	Cell parameters from 2343 reflections
*a* = 7.3525 (5) Å	θ = 2.0–25.0°
*b* = 20.9324 (15) Å	µ = 0.26 mm^−^^1^
*c* = 9.2443 (6) Å	*T* = 273 K
β = 107.565 (2)°	Block, colourless
*V* = 1356.41 (16) Å^3^	0.24 × 0.22 × 0.19 mm
*Z* = 4	

### Data collection

**Table:**

Bruker SMART APEX CCD area-detector diffractometer	2343 independent reflections
Radiation source: fine-focus sealed tube	1981 reflections with *I* > 2σ(*I*)
graphite	*R*_int_ = 0.033
φ and ω scan	θ_max_ = 25.0°, θ_min_ = 2.0°
Absorption correction: multi-scan (*SADABS*; Bruker, 2005)	*h* = −8→8
*T*_min_ = 0.833, *T*_max_ = 0.864	*k* = −24→24
10653 measured reflections	*l* = −10→10

### Refinement

**Table:**

Refinement on *F*^2^	Primary atom site location: structure-invariant direct methods
Least-squares matrix: full	Secondary atom site location: difference Fourier map
*R*[*F*^2^ > 2σ(*F*^2^)] = 0.037	Hydrogen site location: inferred from neighbouring sites
*wR*(*F*^2^) = 0.120	H atoms treated by a mixture of independent and constrained refinement
*S* = 1.12	*w* = 1/[σ^2^(*F*_o_^2^) + (0.0631*P*)^2^ + 0.5954*P*] where *P* = (*F*_o_^2^ + 2*F*_c_^2^)/3
2343 reflections	(Δ/σ)_max_ < 0.001
189 parameters	Δρ_max_ = 0.43 e Å^−^^3^
0 restraints	Δρ_min_ = −0.53 e Å^−^^3^

### Special details

**Table:**

Geometry. All e.s.d.'s (except the e.s.d. in the dihedral angle between two l.s. planes) are estimated using the full covariance matrix. The cell e.s.d.'s are taken into account individually in the estimation of e.s.d.'s in distances, angles and torsion angles; correlations between e.s.d.'s in cell parameters are only used when they are defined by crystal symmetry. An approximate (isotropic) treatment of cell e.s.d.'s is used for estimating e.s.d.'s involving l.s. planes.
Refinement. Refinement of *F*^2^ against ALL reflections. The weighted *R*-factor *wR* and goodness of fit *S* are based on *F*^2^, conventional *R*-factors *R* are based on *F*, with *F* set to zero for negative *F*^2^. The threshold expression of *F*^2^ > σ(*F*^2^) is used only for calculating *R*-factors(gt) *etc*. and is not relevant to the choice of reflections for refinement. *R*-factors based on *F*^2^ are statistically about twice as large as those based on *F*, and *R*- factors based on ALL data will be even larger.

### Fractional atomic coordinates and isotropic or equivalent isotropic displacement parameters (Å^2^)

**Table:**

	*x*	*y*	*z*	*U*_iso_*/*U*_eq_	
C1	0.5180 (5)	0.38868 (12)	0.1480 (3)	0.0457 (7)	
H1	0.4780	0.3769	0.0463	0.055*	
C2	0.5608 (5)	0.45147 (13)	0.1899 (3)	0.0598 (9)	
H2	0.5523	0.4810	0.1131	0.072*	
C3	0.6284 (4)	0.42875 (11)	0.4392 (3)	0.0339 (6)	
H3	0.6653	0.4420	0.5399	0.041*	
C4	0.5927 (3)	0.36459 (11)	0.4100 (2)	0.0297 (5)	
H4	0.6065	0.3359	0.4894	0.036*	
C5	0.5361 (3)	0.34348 (10)	0.2611 (2)	0.0257 (5)	
C6	0.4884 (3)	0.27532 (10)	0.2125 (2)	0.0253 (5)	
C7	0.4594 (3)	0.13874 (10)	0.5560 (3)	0.0289 (5)	
C8	0.4371 (3)	0.19677 (11)	0.6218 (3)	0.0322 (5)	
H8	0.3844	0.2317	0.5616	0.039*	
C9	0.4947 (4)	0.20161 (12)	0.7788 (3)	0.0370 (6)	
H9	0.4826	0.2403	0.8246	0.044*	
C10	0.5702 (4)	0.14896 (14)	0.8673 (3)	0.0424 (6)	
H10	0.6066	0.1523	0.9725	0.051*	
C11	0.5917 (4)	0.09153 (13)	0.8006 (3)	0.0439 (7)	
H11	0.6426	0.0565	0.8611	0.053*	
C12	0.5379 (4)	0.08587 (12)	0.6439 (3)	0.0359 (6)	
H12	0.5540	0.0475	0.5986	0.043*	
N1	0.6133 (3)	0.47292 (9)	0.3323 (2)	0.0400 (5)	
N2	0.5685 (3)	0.23082 (8)	0.31946 (19)	0.0258 (4)	
H2N	0.6398	0.2425	0.4075	0.031*	
N3	0.5352 (3)	0.16619 (9)	0.2867 (2)	0.0293 (5)	
H3N	0.5925	0.1458	0.2324	0.035*	
O1	0.3873 (2)	0.26165 (8)	0.08506 (16)	0.0340 (4)	
O2	0.2057 (2)	0.16569 (8)	0.30300 (19)	0.0366 (4)	
O3	0.3881 (3)	0.06518 (8)	0.31658 (19)	0.0421 (5)	
S1	0.38198 (8)	0.13081 (3)	0.35666 (6)	0.0294 (2)	
H1E	0.942 (6)	0.1209 (16)	0.248 (4)	0.063 (12)*	
H1F	0.849 (5)	0.0671 (18)	0.196 (4)	0.063 (10)*	
O1W	0.8432 (3)	0.10382 (10)	0.2345 (2)	0.0421 (5)	

### Atomic displacement parameters (Å^2^)

**Table:**

	*U*^11^	*U*^22^	*U*^33^	*U*^12^	*U*^13^	*U*^23^
C1	0.074 (2)	0.0366 (14)	0.0207 (12)	−0.0022 (13)	0.0047 (12)	0.0032 (10)
C2	0.109 (3)	0.0334 (15)	0.0298 (14)	−0.0045 (16)	0.0109 (16)	0.0109 (11)
C3	0.0439 (15)	0.0309 (13)	0.0245 (11)	0.0031 (10)	0.0068 (11)	−0.0011 (10)
C4	0.0356 (14)	0.0298 (13)	0.0210 (11)	0.0041 (10)	0.0047 (10)	0.0035 (9)
C5	0.0239 (12)	0.0290 (12)	0.0223 (11)	0.0043 (9)	0.0042 (9)	0.0018 (9)
C6	0.0226 (12)	0.0323 (12)	0.0192 (11)	0.0027 (9)	0.0037 (9)	0.0000 (9)
C7	0.0306 (13)	0.0260 (12)	0.0285 (12)	−0.0040 (9)	0.0064 (10)	−0.0012 (9)
C8	0.0360 (14)	0.0282 (12)	0.0348 (12)	−0.0019 (10)	0.0144 (11)	0.0008 (10)
C9	0.0384 (15)	0.0392 (14)	0.0378 (13)	−0.0110 (11)	0.0181 (12)	−0.0107 (11)
C10	0.0422 (16)	0.0572 (17)	0.0273 (12)	−0.0089 (13)	0.0096 (11)	−0.0029 (12)
C11	0.0493 (18)	0.0434 (16)	0.0350 (14)	0.0029 (12)	0.0068 (12)	0.0098 (12)
C12	0.0393 (15)	0.0298 (13)	0.0341 (13)	0.0012 (11)	0.0044 (11)	0.0001 (10)
N1	0.0537 (14)	0.0300 (11)	0.0331 (11)	0.0006 (9)	0.0084 (10)	0.0014 (9)
N2	0.0283 (11)	0.0243 (10)	0.0191 (9)	−0.0005 (7)	−0.0015 (8)	−0.0017 (7)
N3	0.0354 (12)	0.0252 (10)	0.0259 (9)	0.0027 (8)	0.0073 (8)	−0.0044 (8)
O1	0.0341 (10)	0.0392 (10)	0.0205 (8)	0.0009 (7)	−0.0041 (7)	−0.0024 (7)
O2	0.0273 (10)	0.0362 (10)	0.0401 (10)	−0.0004 (7)	0.0008 (7)	0.0024 (7)
O3	0.0606 (13)	0.0233 (9)	0.0349 (9)	−0.0036 (8)	0.0031 (9)	−0.0048 (7)
S1	0.0332 (4)	0.0227 (3)	0.0273 (3)	−0.0019 (2)	0.0014 (2)	−0.0017 (2)
O1W	0.0352 (12)	0.0344 (11)	0.0582 (12)	−0.0005 (9)	0.0161 (10)	−0.0123 (9)

### Geometric parameters (Å, °)

**Table:**

C1—C2	1.379 (4)	C8—H8	0.9300
C1—C5	1.386 (3)	C9—C10	1.385 (4)
C1—H1	0.9300	C9—H9	0.9300
C2—N1	1.333 (3)	C10—C11	1.382 (4)
C2—H2	0.9300	C10—H10	0.9300
C3—N1	1.333 (3)	C11—C12	1.386 (3)
C3—C4	1.379 (3)	C11—H11	0.9300
C3—H3	0.9300	C12—H12	0.9300
C4—C5	1.385 (3)	N2—N3	1.392 (2)
C4—H4	0.9300	N2—H2N	0.8600
C5—C6	1.505 (3)	N3—S1	1.635 (2)
C6—O1	1.223 (3)	N3—H3N	0.8600
C6—N2	1.356 (3)	O2—S1	1.4393 (17)
C7—C8	1.390 (3)	O3—S1	1.4272 (17)
C7—C12	1.392 (3)	O1W—H1E	0.78 (4)
C7—S1	1.764 (2)	O1W—H1F	0.86 (4)
C8—C9	1.387 (3)		
			
C2—C1—C5	118.5 (2)	C10—C9—H9	120.0
C2—C1—H1	120.8	C8—C9—H9	120.0
C5—C1—H1	120.8	C11—C10—C9	120.5 (2)
N1—C2—C1	124.9 (2)	C11—C10—H10	119.7
N1—C2—H2	117.6	C9—C10—H10	119.7
C1—C2—H2	117.6	C10—C11—C12	120.4 (2)
N1—C3—C4	124.2 (2)	C10—C11—H11	119.8
N1—C3—H3	117.9	C12—C11—H11	119.8
C4—C3—H3	117.9	C11—C12—C7	118.7 (2)
C3—C4—C5	119.2 (2)	C11—C12—H12	120.7
C3—C4—H4	120.4	C7—C12—H12	120.7
C5—C4—H4	120.4	C2—N1—C3	115.6 (2)
C4—C5—C1	117.6 (2)	C6—N2—N3	120.00 (17)
C4—C5—C6	124.90 (19)	C6—N2—H2N	120.0
C1—C5—C6	117.5 (2)	N3—N2—H2N	120.0
O1—C6—N2	123.1 (2)	N2—N3—S1	116.82 (14)
O1—C6—C5	121.90 (19)	N2—N3—H3N	121.6
N2—C6—C5	115.02 (18)	S1—N3—H3N	121.6
C8—C7—C12	121.5 (2)	O3—S1—O2	119.65 (11)
C8—C7—S1	119.73 (18)	O3—S1—N3	104.70 (10)
C12—C7—S1	118.79 (17)	O2—S1—N3	106.88 (10)
C9—C8—C7	118.9 (2)	O3—S1—C7	109.64 (10)
C9—C8—H8	120.6	O2—S1—C7	106.57 (11)
C7—C8—H8	120.6	N3—S1—C7	109.05 (10)
C10—C9—C8	120.1 (2)	H1E—O1W—H1F	108 (3)

### Hydrogen-bond geometry (Å, °)

**Table:**

*D*—H···*A*	*D*—H	H···*A*	*D*···*A*	*D*—H···*A*
N3—H3N···O1W	0.86	2.04	2.779 (3)	144
O1W—H1E···O2^i^	0.78 (4)	2.07 (4)	2.857 (3)	175 (4)

Symmetry codes:  (i) *x*+1, *y*, *z*.

## References

[bb1] Bruker (2002). *SMART* and *SAINT* Bruker AXS, Inc., Madison, Wisconsin, USA.

[bb2] Bruker (2005). *SADABS* Bruker AXS, Inc., Madison, Wisconsin, USA.

[bb3] Farrugia, L. J. (1997). *J. Appl. Cryst.* **30**, 565.

[bb4] Farrugia, L. J. (1999). *J. Appl. Cryst.* **32**, 837–838.

[bb5] Lemin, A. J. (1961). US Patent 2993829.

[bb6] Shanbhag, A. V., Venkatesha, T. V., Prabhu, R. A., Kalkhambkar, R. G. & Kulkarni, G. M. (2008). *J. Appl. Electrochem.* **38**, 279–287.

[bb7] Sheldrick, G. M. (2008). *Acta Cryst.* A**64**, 112–122. 10.1107/S0108767307043930 18156677

[bb8] Zhen, X.-L. & Li, X.-L. (2008). *Acta Cryst.* E**64**, o2170. 10.1107/S1600536808034089 PMC2959596 21581030

